# Prognostic Value of Long Noncoding RNA SNHG12 in Various Carcinomas: A Meta-Analysis

**DOI:** 10.1155/2020/8847401

**Published:** 2020-11-26

**Authors:** Zhi-Ran Li, Qin Yang, Tao Zhou, Yan-Hua Huang, Hua-Zhu Zhang, Rui Meng, Gong-Hao He

**Affiliations:** ^1^Research Center of Clinical Pharmacology, Yunnan Provincial Hospital of Traditional Chinese Medicine, Kunming 650021, China; ^2^Department of Clinical Pharmacy, 920th Hospital of Joint Logistics Support Force, Kunming 650032, China

## Abstract

**Background:**

Numerous recent studies suggested that overexpression of the long noncoding RNA small nucleolar RNA host gene 12 (SNHG12) exhibited prooncogenic activity in multiple cancers. However, results regarding the prognostic value of SNHG12 in cancers still remained controversial. Therefore, we conducted a meta-analysis complemented with bioinformatics analysis to elucidate the clinical significance of SNHG12 in cancer patients.

**Methods:**

PubMed, Embase, Cochrane Library, Chinese National Knowledge Infrastructure, Wanfang, and Weipu databases were searched for eligible studies until July 2020. Additionally, bioinformatics analysis was applied to verify the results of meta-analysis.

**Results:**

Twenty-three related studies consisting of 1389 cancer patients were enrolled in the current meta-analysis. Elevated SNHG12 expression was found to be significantly associated with poor overall survival (OS) (HR = 1.81; 95% CI: 1.53-2.13; *P* < 0.001) and disease-free survival (DFS) (HR = 1.40; 95% CI: 1.12-1.76; *P* = 0.004) in multiple cancers, which were also verified by the results of bioinformatics analysis. Moreover, overexpression of SNHG12 was also related to clinicopathological characteristics including LNM, distant metastasis, high clinical stage, large tumor size, and poor tumor differentiation in diverse types of cancers.

**Conclusion:**

The present findings indicated that SNHG12 might act as a novel biomarker for diagnosis or prognosis in human cancers.

## 1. Introduction

Cancer is a heterogeneous disease with increasing incidence and mortality worldwide, which is also considered as a major barrier to increasing life expectancy [1]. Despite substantial advances in diagnosis and treatment of cancer patients, the five-year survival rate in diverse cancer types is still significantly poor, mainly owing to the fact that many cancer patients are diagnosed at an advanced stage [2]. Thus, identification of promising diagnostic and prognostic biomarkers in early stages is critical to improve the survival status of cancer patients.

Long noncoding RNAs (lncRNAs) are a class of nonprotein-coding RNAs with >200 nucleotides in length [3], which were previously found to play vital roles in various biological activities, such as genomic regulation and cell cycle regulation [4, 5]. During these physiological and/or pathophysiological processes, lncRNAs act as not only oncogenes but also tumor suppressor genes from the functional point of view [6]. As a result, functional lncRNAs attracted wide attention as diagnostic/prognostic biomarkers or therapeutic targets in malignant tumors [7, 8]. Therefore, identifying the clinical effects of certain lncRNA tightly correlated with cell malignant transformation and tumor progression would eventually provide potential ways for prevention and treatment of corresponding cancers.

Small nucleolar RNA host gene 12 (SNHG12) is a lncRNA located at chromosome 1p35.3 and was originally reported to be overexpressed in endometrial cancer [9, 10]. Recently, increasing evidence showed that SNHG12 was also upregulated and contributed to tumor proliferation, invasion, and migration in various tumor tissues, including cervical [11, 12], gastric [13–16], ovarian [17], renal [18], laryngeal [19], lung [20], hepatocellular [21, 22], colorectal [23, 24], prostate [25], nasopharyngeal [26], and breast cancer [27], as well as glioma [28, 29], osteosarcoma [30, 31], and diffuse large B cell lymphoma (DLBCL) [32]. According to previous fundamental studies, SNHG12 had a series of ways to affect the tumorigenesis and development of these cancers. For instance, it was reported that SNHG12 mediated tumor immune escape through its involvement in unfolded protein responses [33]. Furthermore, SNHG12 was found to promote IL-6/miR-21 crosstalk between tumor cells and M2 macrophages and facilitate cancer progression [34]. Additionally, SNHG12 was also reported to facilitate cancer growth by activating various signaling pathways [16, 21], promoting epithelial-mesenchymal transition (EMT) [20], or serving as competing endogenous RNAs (ceRNAs) [14, 17]. In accordance with these findings, recent clinical investigations further indicated that increased SNHG12 expression was correlated with worse clinicopathologic features and poor survival [11–16]. Nevertheless, most individual clinical studies on SNHG12 were performed with relatively small samples. In addition, inconsistent results were also obtained in studies on the associations between SNHG12 expression and clinical features such as age [26, 28], clinical stage [21, 27], tumor size [22, 28], tumor differentiation [11, 26], lymph node metastasis [13, 16], and distant metastasis [13, 16]. Therefore, there is still much uncertainty for the prognostic value of SNHG12 in cancers, and a systematic analysis is still needed to clarify this issue, which, however, has not been explored so far as we know.

Based on the above background, a comprehensive meta-analysis was performed to elucidate the expression status and clinical value of SNHG12 in cancer patients. The results of the current study may contribute to illuminating the potential predictive value of SNHG12 in human cancers.

## 2. Materials and Methods

### 2.1. Literature Search

A comprehensive search was performed in PubMed, Embase, Cochrane Library, Chinese National Knowledge Infrastructure, Wanfang, and Weipu databases to obtain all relevant studies up to July 23, 2020. The keywords were as follows: “long non-coding RNA small nucleolar RNA host 12,” “long non-coding RNA SNHG12” “SNHG12 lncRNA” “lncRNA SNHG12” or “SNHG12,” “carcinoma” or “tumor” or “neoplasm” or “cancer.” The electronic search strategy for PubMed was provided in Supplementary Table S1.

### 2.2. Selection Criteria

The inclusion criteria are as follows: (1) the level of SNHG12 expression was detected in any malignant tumor, (2) the relationship between SNHG12 expression and prognosis was investigated, and (3) sufficient data for calculating the hazard ratios (HRs) with 95% confidence intervals (CIs). By contrast, the following literatures were excluded: (1) reviews, conference abstracts, and letters; (2) nonhuman trials; (3) the data from The Cancer Genome Atlas (TCGA).

### 2.3. Data Extraction and Quality Assessment

The data of each eligible publication was extracted by two investigators independently. Any disagreements were discussed with a third investigator. Baseline data acquisition included: author, publication year, country, sample size, cancer type, follow-up interval, cut-off value, detection method, HR and 95% CI, survival analysis method, and Newcastle-Ottawa scale (NOS) score. If HRs and 95% CIs were obtained from univariate and multivariate analysis, the latter was the priority. If HRs were not directly accessible in the text, the survival rates were extracted from the survival curves using Engauge Digitizer 4.1 [35].

The NOS scoring system with scores ranging from 0 to 9 points was utilized to evaluate the quality of eligible studies. Studies with a cumulative score of more than 5 points were identified as high quality in methodology to include in the meta-analysis.

### 2.4. Ethical Statement

The ethical approval or patient consent was not required since all data were based on already published literatures.

### 2.5. Statistical Analysis

All data syntheses and graphic plotting were done with STATA 12.0. Results with *P* < 0.05 were regarded as statistically significant. The pooled HRs and pooled odds ratios (ORs) were calculated to assess the correlation of SNHG12 expression with prognosis and clinic-pathological parameters in cancers, respectively. Heterogeneity between studies was assessed via *I*^2^ statistics and the *Q* test. The random-effects model was applied when heterogeneity was statistically significant (*P* < 0.05 and *I*^2^ > 50%). Otherwise, the fixed-effects model was utilized. In addition, the stability of consequences was assessed through sensitivity analysis. Begg's and Egger's tests were performed for publication bias assessment if the number of enrolled studies ≥10 [36, 37]. When such bias existed, the trim-and-fill method was used for correcting pooled result as previously described [38].

### 2.6. Bioinformatics Analysis

Gene Expression Profiling Interactive Analysis (GEPIA) was used to verify SNHG12 expression levels in different types of cancers and its correlations with OS/DFS based on the data from TCGA and GTEx datasets [39]. Additionally, correlation analysis between the expression levels of two genes was also performed using the data from TCGA [39].

## 3. Results

### 3.1. Study Selection

**Figure 1:**
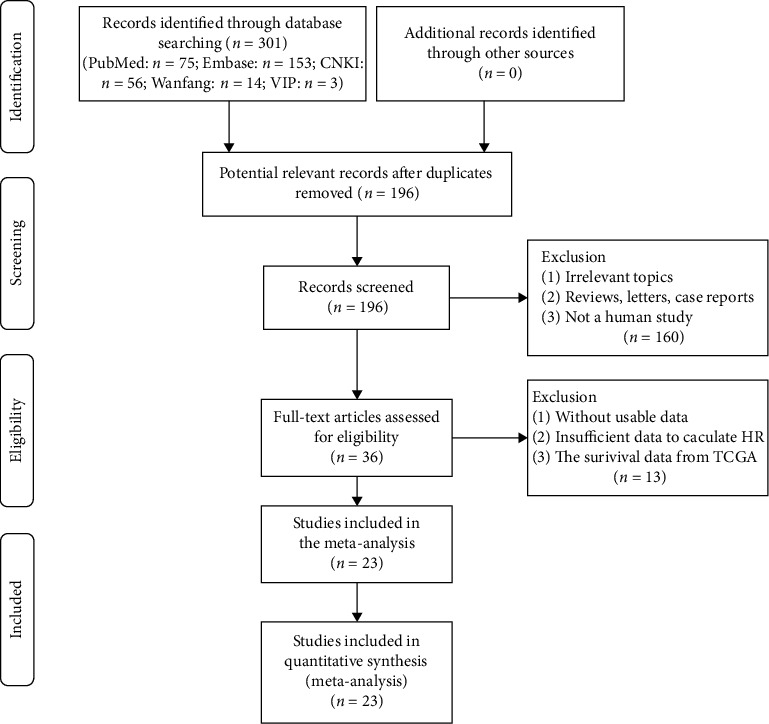
Flow diagram of study selection procedure.

This meta-analysis was performed in accordance with the Preferred Reporting Items for Systematic Reviews and Meta-Analyses (PRISMA) statement (Supplementary Table S2). Initially, 196 articles were obtained after excluding duplicate publications. Furthermore, after excluding 160 unrelated articles by screening the title and abstract, the remaining studies were further reviewed of the full texts and 13 publications were excluded, which did not provide available data. Finally, 23 articles were enrolled into this meta-analysis (Figure 1).

### 3.2. Study Characteristics

The basic information of the 23 enrolled articles were listed in Table 1. A total of 1389 patients were included, and the mean patient sample size for each study was 60 (range, 20-129). Fifteen different types of cancers were analyzed, including gastric cancer (GC) (*n* = 4), cervical cancer (CC) (*n* = 2), hepatocellular carcinoma (HCC) (*n* = 2), glioma (*n* = 2), colorectal cancer (CRC) (*n* = 2), osteosarcoma (*n* = 2), DLBCL (*n* = 1), nasopharyngeal carcinoma (NPC) (*n* = 1), ovarian cancer (OC) (*n* = 1), renal cell carcinoma (RCC) (*n* = 1), esophageal squamous cell cancer (ESCC) (*n* = 1), non-small-cell lung cancer (NSCLC) (*n* = 1), prostate cancer (PCa) (*n* = 1), laryngeal squamous cell carcinoma (LSCC) (*n* = 1), and breast cancer (BC) (*n* = 1). All studies were of high quality with their NOS scores ≥ 5 (range, 5-9).

### 3.3. SNHG12 and Main Survival Outcome

**Figure 2:**
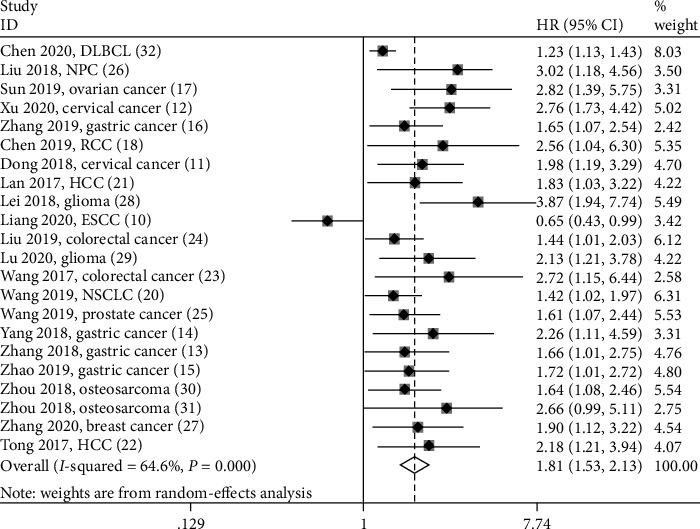
Forest plots for the association between SNHG12 expressions with overall survival.

**Figure 3:**
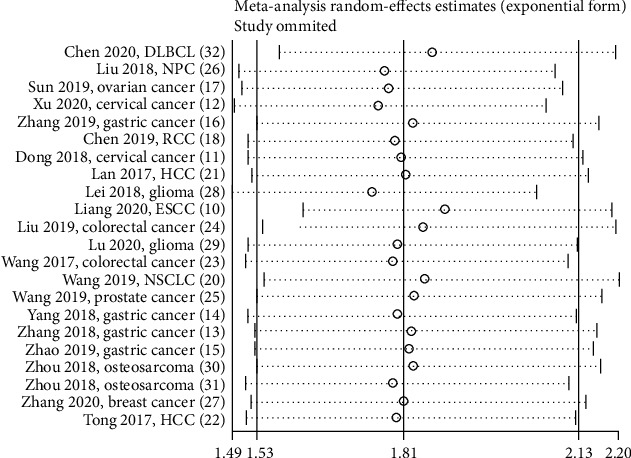
Sensitivity analysis on the relationship between SNHG12 expression and overall survival.

**Figure 4:**
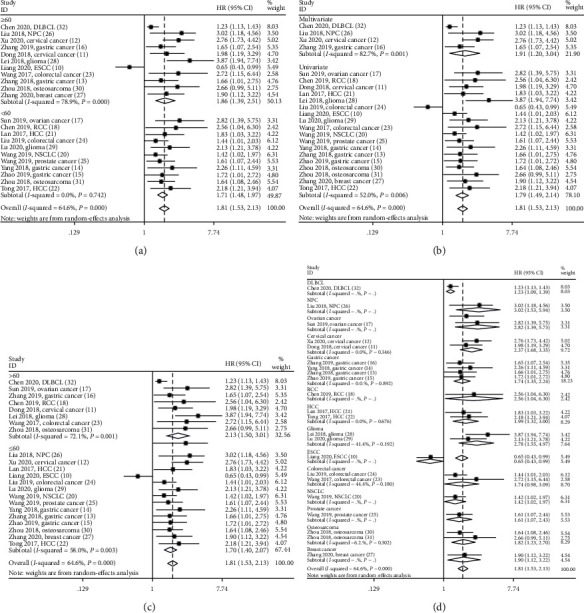
Forest plots of the subgroup analysis evaluating hazard ratios (HRs) of SNHG12 for overall survival by the factors of (a) sample size, (b) survival analysis method, (c) follow-up time, and (d) cancer type.

Twenty-two studies consisting of 1339 cancer patients provided overall survival (OS) data. As shown in Figure 2, elevated SNHG12 expression was strongly related to poor OS in multiple cancers (HR = 1.81; 95% CI: 1.53-2.13; *P* < 0.001). Because of heterogeneity (*I*^2^ = 64.6% and *P* < 0.001), sensitivity analysis was conducted to assess the stability of the pooled result between SNHG12 expression and OS. After each single study was removed alternately, the pooled result was not remarkably changed (Figure 3). Then, subgroup meta-analyses stratified by sample size (<60 and ≥60), survival analysis method (univariate and multivariate), and follow-up months (≤60 and >60) were also performed. We found a significant correlation of increased SNHG12 expression with poorer OS in all above factors (Figures 4(a)–4(c) and Table 2). Moreover, subgroup analyses based on cancer type were performed to maximize clinical relevance. The overall results showed that increased SNHG12 expression was associated with shorter OS in GC (HR = 1.74; 95% CI: 1.35-2.24; *P* < 0.001), CC (HR = 2.37; 95% CI: 1.68-3.35; *P* < 0.001), HCC (HR = 1.99; 95% CI: 1.32-3.00; *P* = 0.001), glioma (HR = 2.78; 95% CI: 1.55-4.97; *P* = 0.001), osteosarcoma (HR = 1.82; 95% CI: 1.23-2.70; *P* = 0.003), DLBCL (HR = 1.23; 95% CI: 1.09-1.39; *P* = 0.001), NPC (HR = 3.02; 95% CI: 1.53-5.94; *P* = 0.001), OC (HR = 2.82; 95% CI: 1.39-5.73; *P* = 0.004), RCC (HR = 2.56; 95% CI: 1.04-6.30; *P* = 0.041), NSCLC (HR = 1.42; 95% CI: 1.02-1.97; *P* = 0.037), PCa (HR = 1.61; 95% CI: 1.07-2.43; *P* = 0.024), and BC (HR = 1.90; 95% CI: 1.12-3.22; *P* = 0.017) (Figure 4(d)). However, we observed that patients with low SNHG12 expression had poor OS in ESCC (HR = 0.65; 95% CI: 0.43-0.99; *P* = 0.043) (Figure 4(d)). Of note, the heterogeneity was diminished significantly in the individual cancer types, suggesting that the cancer type was probably the major source of heterogeneity.

**Figure 5:**
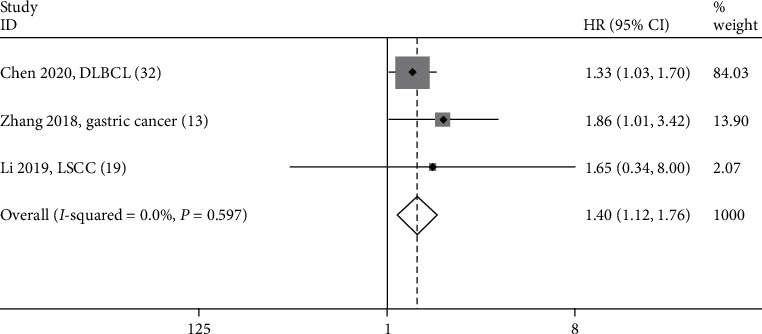
Forest plots for the association between SNHG12 expressions with disease-free survival.

Three studies with 190 subjects investigated the relationship between SNHG12 expression and disease-free survival (DFS) in patients with GC, LSCC, and DLBCL [13, 19, 32]. The result demonstrated that overexpression of SNHG12 also predicted poor DFS in cancers (HR = 1.40; 95% CI: 1.12-1.76; *P* = 0.004) (Figure 5). A fixed-effects model was applied because that no apparent heterogeneity was observed (*I*^2^ = 0.0% and *P* = 0.597). In addition, only one study focused on the association between SNHG12 expression and recurrence-free survival (RFS), which suggested that increased SNHG12 expression was strongly associated with poor RFS in HCC [21].

### 3.4. Association between SNHG12 and Clinicopathological Characteristics

**Figure 6:**
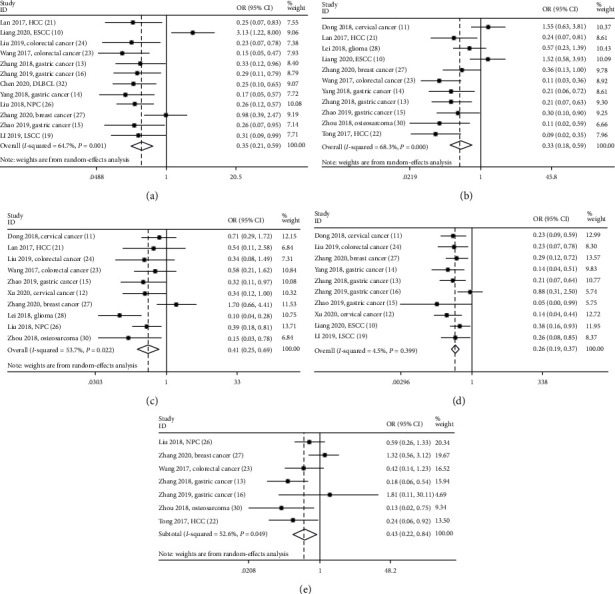
Forest plots of studies evaluating odds ratios (ORs) of SNHG12 **e**xpression and the clinicopathology features, including (a) clinical stage, (b) tumor size, (c) differentiation grade, (d) lymph node metastasis, (e) distant metastasis.

As presented in Table 3 and Figure 6, metaresults showed that elevated SNHG12 expression was found to be closely related to advanced clinical stage (OR = 0.35; 95% CI: 0.21-0.59; *P* < 0.001), larger tumor size (OR = 0.33; 95% CI: 0.18-0.59; *P* < 0.001), poor tumor differentiation (OR = 0.41; 95% CI: 0.25-0.69; *P* = 0.001), lymph node metastasis (LNM) (OR = 0.26; 95% CI: 0.19-0.37; *P* < 0.001), and distant metastasis (OR = 0.43; 95% CI: 0.22-0.84; *P* = 0.014). Nevertheless, no apparent association was found between overexpression of SNHG12 and age (*P* = 0.403), gender (*P* = 0.291), tumor number (*P* = 0.586), and vascular invasion (*P* = 0.828) (Supplementary Figure S1).

### 3.5. Publication Bias

**Figure 7:**
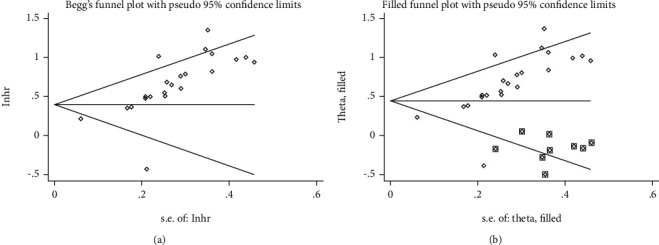
Publication bias of SNHG12 expression for overall survival. (a) Begg's funnel plot; (b) filled funnel plot after adjustment by using the “trim-and-fill” method.

We performed publication bias for OS and clinicopathological characteristics including age, gender, clinical stage, LNM, tumor differentiation, and tumor size. Both Begg's funnel diagram and Egger's test indicated that no obvious publication bias for age (*P* = 0.875), gender (*P* = 0.194), clinical stage (*P* = 0.296), LNM (*P* = 0.118), or tumor differentiation (*P* = 0.479) (Supplementary Figure S2a–e). However, there was publication bias in tumor size (*P* = 0.003) (Supplementary Figure S2f) and OS (*P* < 0.001) (Figure 7(a)). Therefore, we applied the trim-and-fill method, and there were no missing trials trimmed in the tumor size. As for OS, after filling nine trials (Figure 7(b)), the result was still consistent using fixed model (HR = 1.39; 95% CI: 1.28-1.50; *P* < 0.001) or random model (HR = 1.50; 95% CI: 1.29-1.75; *P* < 0.001), which suggested that there is no publication bias in the comparison.

### 3.6. Validation of the Results in GEPIA

**Figure 8:**
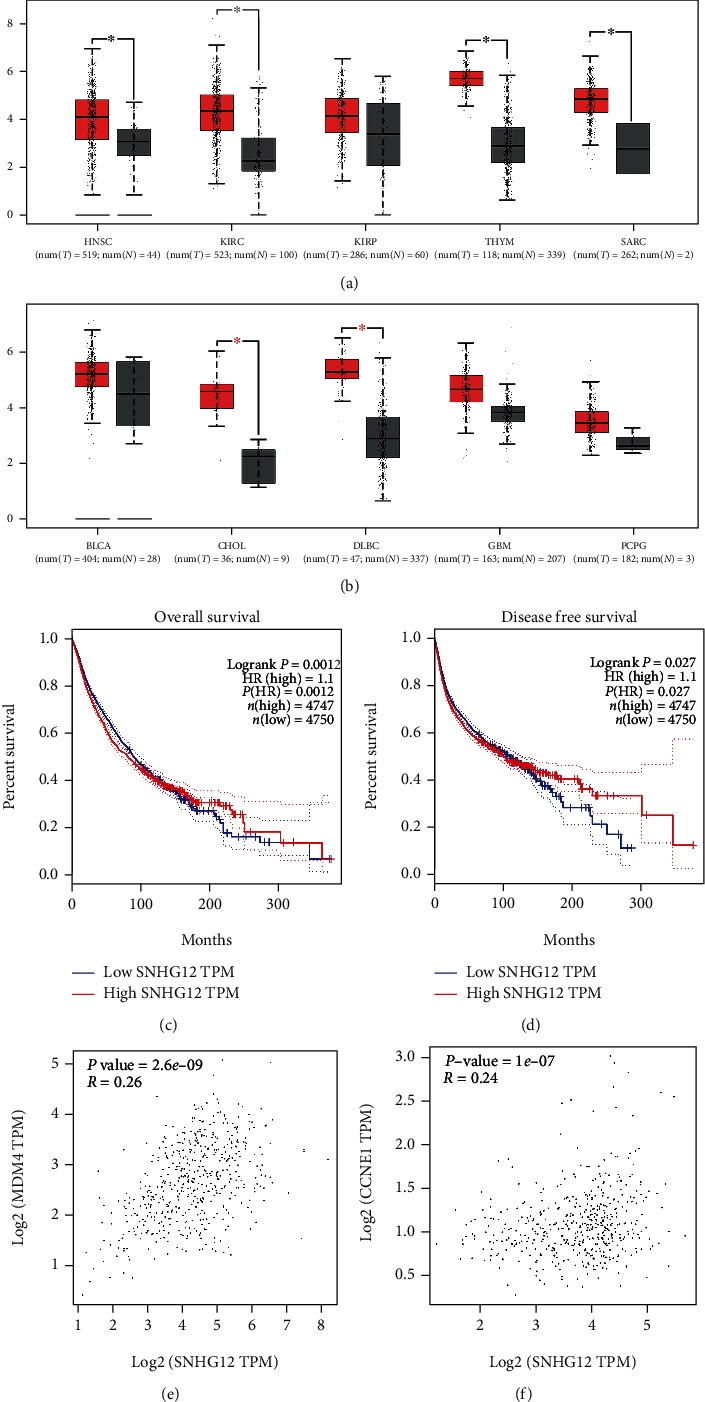
Bioinformatics analysis based on the data from Gene Expression Profiling Interactive Analysis (GEPIA) database. (a, b) The expression levels of SNHG12 between cancerous and normal tissues in patients within head and neck squamous cell carcinoma (HNSC), kidney renal clear cell carcinoma (KIRC), kidney renal papillary cell carcinoma (KIRP), thymoma (THYM), sarcoma (SARC), bladder urothelial carcinoma (BLCA), cholangiocarcinoma (CHOL), lymphoid neoplasm diffuse large B cell lymphoma (DLBC), glioblastoma multiforme (GBM), and pheochromocytoma and paraganglioma (PCPG). (c) Overall survival plot and (d) disease-free survival plot of SNHG12 in various cancer patients. (e) Correlation between SNHG12 and MDM4 expression in KIRC. (f) Correlation between SNHG12 and cyclin E1 (CCNE1) in prostate adenocarcinoma (PRAD).

As shown in Figures 8(a) and 8(b), SNHG12 expression was higher in a majority of tumor tissues than in normal tissues. In addition, the pooled results (Figures 8(c) and 8(d)) of survival analysis in various malignancies showed that SNHG12 overexpression predicted worse OS/DFS, which strengthen the results of our meta-analysis. Besides, correlation analysis showed a positive collection between the expression of SNHG12 and MDM4 in kidney renal clear cell carcinoma (KIRC) (Figure 8(e)). SNHG12 and cyclin E1 (CCNE1) expression levels were also positively correlated in prostate adenocarcinoma (PRAD) (Figure 8(f)).

## 4. Discussion

This is the first systematic meta-analysis for evaluating the pooled prognostic value of SNHG12 in human cancers to the best of our knowledge. A total of 23 studies with 15 different types of cancers comprising 1389 cancer patients were enrolled in this study. The results showed that SNHG12 might be an unfavorable prognosis factor for cancer patients since high SNHG12 expression was strongly related to shorter survival and poor clinical features, which was in accordance with most of the previous findings that SNHG12 exhibits prooncogenic activity in vitro and in vivo experiments. The current findings will contribute to the further knowledge of SNHG12 as an effective diagnostic or prognostic biomarker and also provide valuable information for cancer therapy.

The potential mechanisms underlying the relationship between aberrant SNHG12 expression and poor clinical prognosis in cancers were well-studied previously. Accumulating studies revealed that SNHG12 served as a ceRNA, similar to miRNA “sponge,” to modulate multiple cancer-related pathophysiological processes. It was reported that SNHG12 accelerated the progression of gastric carcinoma by regulating Argo2 expression via sponging miR-199a/b-5p [14]. Furthermore, it was also showed that SNHG12 exerted its carcinogenic effects by interacting with miR-129 and upregulating the expression of SOX4 and thereby promoted ovarian cancer progression [17]. Moreover, SNHG12 was also reported to be involved in the tumorigenesis of other cancers by interacting with miR-129-5p/WWP1, miR-195/CCNE1, miR-125-5p/MDM4, miR-326/E2F1, and miR-15a-5p/SALL4 axes [19, 20, 40–42]. Besides this ability to function as a ceRNA, SNHG12 was further suggested to activate various signaling pathways, including MLK3/I*κ*B/NF-*κ*B pathway, Notch-1 signaling pathway, and PI3K/AKT pathway [16, 21, 26]. Additionally, the results of correlation analysis between SNHG12 and relevant target genes in GEPIA were also consistent with these additional previous fundamental studies, such as that SNHG12 was positively related with MDM4 (*R* = 0.26 and *P* = 2.6*e* − 09) and CCNE1 (*R* = 0.24 and *P* = 1*e* − 07), respectively. Taken together, these mechanisms indicated that SNHG12 acted as important regulators in the progress of cancers and further supported our findings that increased SNHG12 expression predicted poor OS in cancer patients.

Besides, it should be noted that certain previous study employed serum samples to detect endogenous SNHG12 expression levels with relatively good diagnostic efficiency in specific cancer [25]. As detecting the expression levels of SNHG12 in blood is relatively easy to operate with minimal trauma, this lncRNA would be more suitable as a biomarker for clinical application than those that need to be detected by biopsy although its diagnostic value in blood for other cancer types still needed to be further verified.

Additionally, the results of our further subgroup analyses based on cancer types indicated that enhanced expression of SNHG12 was positively associated with better prognosis in ESCC. This finding was convincing as it was previously demonstrated that knockdown SNHG12 significantly boosted cellular growth and promoted cell migration in ESCC and that SNHG12/miRNA-195-5p/BCL9 network may be implicated in ESCC progression [10]. However, since only one study with relatively small sample size investigated the role of SNHG12 in ESCC, further studies based on large sample size are still needed to clarify this issue. Anyway, this result indicated that the function of SNHG12 would be different in diverse cancer types as a result of different interaction mechanisms and participating partners. However, although the underlying mechanisms of SNHG12 varied with cancer types, the associations of this lncRNA with the abovementioned human cancers still remained significant according to the present analyses, further demonstrating its prognostic value for certain cancers. Moreover, these differential mechanisms also made SNHG12 a potential therapeutic target for respective treatment of corresponding cancer types as well.

Accumulating reports also provided evidence that SNHG12 exerted its metastatic properties through different mechanisms [20, 26, 30, 43]. For instance, it was demonstrated that SNHG12 promoted EMT by regulating the expression of genes involved in EMT (i.e., E-cadherin, vimentin, and N-cadherin) and then contributed to NPC cell migration and invasion [26]. Furthermore, SNHG12 suppressed miR-218 expression and thereby accelerated NSCLC cell metastasis by inducing EMT via the Slug/ZEB2 signaling pathway [20]. Besides, it was also reported that SNHG12 facilitated papillary thyroid carcinoma cell migration and invasion partly by regulating Wnt/*β*-catenin pathway [43]. In addition, upregulation of SNHG12 contributed to migration abilities by facilitating cell cycle progression at the G0/G1 phase [30]. In accordance with these abovementioned mechanisms, current metaresults revealed that elevated SNHG12 expression was closely related to LNM and distant metastasis. Moreover, as distant metastasis is an important reason for failure of cancer therapy, SNHG12 might also serve as a potential therapeutic target for preventing metastasis in diverse cancer types.

Additionally, several limitations in present meta-analysis should be acknowledged. First, HRs and 95% CIs for some studies were obtained from survival curves, which may lead to a calculation bias. Second, the sample size for each type of cancer was relatively small and many factors, such as cut-off value, tumor size, treatment strategy, and concomitant disease, were different in each study, which may lead to underpowered or false positive results. Finally, all of the selected articles were based on Chinese participants; thus, the prognostic value of SNHG12 for other ethnic groups still needed to be further investigated. These limitations should be noticed and addressed in future clinical investigations.

In conclusion, the results of current meta- and bioinformatics analyses showed that overexpression of SNHG12 was significantly related to unfavorable survival outcome and aggressive clinical characteristics including LNM, distant metastasis, high clinical stage, large tumor size, and poor tumor differentiation in multiple cancers, which suggested that SNHG12 might act as a promising diagnostic or prognostic biomarker in cancer patients. Future studies with larger sample size are still needed to verify the clinical significance of SNHG12 in various cancers of different ethnic populations.

## Figures and Tables

**Table 1:** The main information of included studies in the meta-analysis.

Author, year	Country	Cancer	Sample size	Cut-off value	Follow-up (months)	Detection method	Survival analysis	Outcome measure	NOS score
Chen 2020 (32)	China	DLBCL	80	Median	80	qRT-PCR	Multivariate	OS/DFS	9
Liu 2018 (26)	China	NPC	129	Median	60	RT-PCR	Multivariate	OS	9
Xu 2020 (12)	China	Cervical cancer	74	2.25	60	RT-PCR	Multivariate	OS	9
Zhang 2019 (16)	China	Gastric cancer	75	N/A	78	FISH	Multivariate	OS	8
Sun 2019 (17)	China	Ovarian cancer	24	N/A	70	qRT-PCR	Univariate	OS	6
Chen 2019 (18)	China	RCC	20	N/A	108	q-PCR	Univariate	OS	6
Dong 2018 (11)	China	Cervical cancer	76	Mean	80	qRT-PCR	Univariate	OS	7
Lan 2017 (21)	China	HCC	48	Median	48	qPCR	Univariate	OS/RFS	7
Lei 2018 (28)	China	Glioma	79	Median	76	RT-PCR	Univariate	OS	9
Liang 2020 (10)	China	ESCC	85	N/A	50	qRT-PCR	Univariate	OS	6
Liu 2019 (24)	China	Colorectal cancer	53	Mean	60	RT-qPCR	Univariate	OS	7
Lu 2020 (29)	China	Glioma	40	N/A	26	qRT-PCR	Univariate	OS	5
Wang 2017 (23)	China	Colorectal cancer	60	Median	73	qRT-PCR	Univariate	OS	8
Wang 2019 (20)	China	NSCLC	40	Median	48	RT-qPCR	Univariate	OS	7
Wang 2019 (25)	China	Prostate cancer	56	Mean	45	qPCR	Univariate	OS	6
Yang 2018 (14)	China	Gastric cancer	54	N/A	46	qRT-PCR	Univariate	OS	6
Zhang 2018 (13)	China	Gastric cancer	60	Median	60	RT-qPCR	Univariate	OS/DFS	8
Zhao 2019 (15)	China	Gastric cancer	56	Mean	60	RT-qPCR	Univariate	OS	8
Zhou 2018 (30)	China	Osteosarcoma	31	Median	60	qRT-PCR	Univariate	OS	7
Zhou 2018 (31)	China	Osteosarcoma	64	N/A	80	qRT-PCR	Univariate	OS	7
Zhang 2020 (27)	China	Breast cancer	85	Mean	60	qRT-PCR	Univariate	OS	9
Li 2019 (19)	China	LSCC	50	N/A	36	RT-qPCR	Univariate	DFS	5
Tong 2017 (22)	China	HCC	50	Median	60	RT-qPCR	Univariate	OS	8

Abbreviations: DLBCL: diffuse large B cell lymphoma; NPC: nasopharyngeal carcinoma; RCC: renal cell carcinoma; HCC: hepatocellular carcinoma; ESCC: esophageal squamous cell cancer; NSCLC: non-small-cell lung cancer; LSCC: laryngeal squamous cell carcinoma; qRT-PCR: quantitative real-time polymerase chain reaction; FISH: fluorescence in situ hybridization; OS: overall survival; DFS: disease-free survival; RFS: recurrence-free survival.

**Table 2:** Subgroup meta-analysis of pooled hazard ratios for overall survival.

Subgroup analysis	No. of studies	No. of patients	HR (95% CI) random	Significance (*P* value)	Heterogeneity *I*^2^ (%), *P* value
Sample size					
≥60	11	867	1.86 (1.39, 2.51)	<0.001	78.9%, 0.000
<60	11	472	1.71 (1.48, 1.97)	<0.001	0.0%, 0.742

Survival analysis method					
Multivariate	4	382	1.91 (1.20, 3.04)	0.006	82.7%, 0.001
Univariate	18	957	1.79 (1.49, 2.14)	<0.001	52.0%, 0.006

Follow-up (months)					
>60	8	478	2.13 (1.50, 3.01)	<0.001	72.1%, 0.001
≤60	14	861	1.70 (1.40, 2.07)	<0.001	58.0%, 0.003

Tumor type					
Gastric cancer	4	245	1.74 (1.35, 2.24)	<0.001	0.0%, 0.892
Cervical cancer	2	150	2.37 (1.68, 3.35)	<0.001	0.0%, 0.346
HCC	2	98	1.99 (1.32, 3.00)	0.001	0.0%, 0.676
Glioma	2	119	2.78 (1.55, 4.97)	0.001	41.4%, 0.192
Colorectal cancer	2	113	1.74 (0.98, 3.09)	0.057	44.4%, 0.180
Osteosarcoma	2	95	1.82 (1.23, 2.70)	0.003	6.2%, 0.302
DLBCL	1	80	1.23 (1.09, 1.39)	0.001	-
NPC	1	129	3.02 (1.53, 5.94)	0.001	-
Ovarian cancer	1	24	2.82 (1.39, 5.73)	0.004	-
RCC	1	20	2.56 (1.04, 6.30)	0.041	-
ESCC	1	85	0.65 (0.43, 0.99)	0.043	-
NSCLC	1	40	1.42 (1.02, 1.97)	0.037	-
Prostate cancer	1	56	1.61 (1.07, 2.43)	0.024	-
Breast cancer	1	85	1.90 (1.12, 3.22)	0.017	-

Abbreviations: HCC: hepatocellular carcinoma; DLBCL: diffuse large B cell lymphoma; NPC: nasopharyngeal carcinoma; RCC: renal cell carcinoma; ESCC: esophageal squamous cell carcinoma; NSCLC: non-small-cell lung cancer; HR: hazard ratio.

**Table 3:** Association between lncRNA SNHG12 and clinicopathological characteristics of cancer patients.

Clinicopathological parameters	No. of studies	No. of patients	OR (95% CI)	*P* value	Heterogeneity *I*^2^ (%), *P* value	Model
Age (young vs. elder)	18	1206	0.90 (0.72, 1.14)	0.403	3.5%, 0.414	Fixed
Gender (male vs. female)	14	910	1.16 (0.88, 1.51)	0.291	0.0%, 0.543	Fixed
Tumor size (small vs. large)	11	689	0.33 (0.18, 0.59)	<0.001	68.3%, <0.001	Random
Tumor differentiation (well and moderately vs. poorly)	10	696	0.41 (0.25, 0.69)	0.001	53.7%, 0.022	Random
Lymph node metastasis (no vs. yes)	10	673	0.26 (0.19, 0.37)	<0.001	4.5%, 0.399	Fixed
Clinical stage (I–II vs. III–IV)	12	835	0.35 (0.21, 0.59)	<0.001	64.7%, 0.001	Random
Distant metastasis (no vs. yes)	7	490	0.43 (0.22, 0.84)	0.014	52.6%, 0.049	Random
Vascular invasion (no vs. yes)	2	129	0.76 (0.06, 9.32)	0.828	89.9%, 0.002	Random
Tumor number (single vs. multiple)	2	129	0.75 (0.26, 2.12)	0.586	0.0%, 0.826	Fixed

Abbreviations: OR: odds ratio.

## Data Availability

The data supporting the conclusions of this study are available from the corresponding author upon request.

## Supplementary Materials

Supplementary 1 Supplementary Figure S1. Forest plots of studies evaluating odds ratios (ORs) of SNHG12 expression and the clinicopathology features, including (a) age, (b) gender, (c) tumor number, and (d) vascular invasion. Supplementary Figure S2: funnel plots (Begg's method) of potential publication bias for (a) age, (b) gender, (c) clinical stage, (d) lymph node metastasis, (e) differentiation grade, and (f) tumor size.

Supplementary 2 Supplementary Table S1. The detailed example of the full electronic search strategy for PubMed. Supplementary Table S2: PRISMA Checklist.
