# Supplementary figures and images for: Prognostic Value of Long Noncoding RNA SNHG12 in Various Carcinomas: A Meta-Analysis
Source: Biomed Res Int. 2020 Nov 26;2020:8847401. doi: 10.1155/2020/8847401 (PMC7716752; doi:10.1155/2020/8847401)

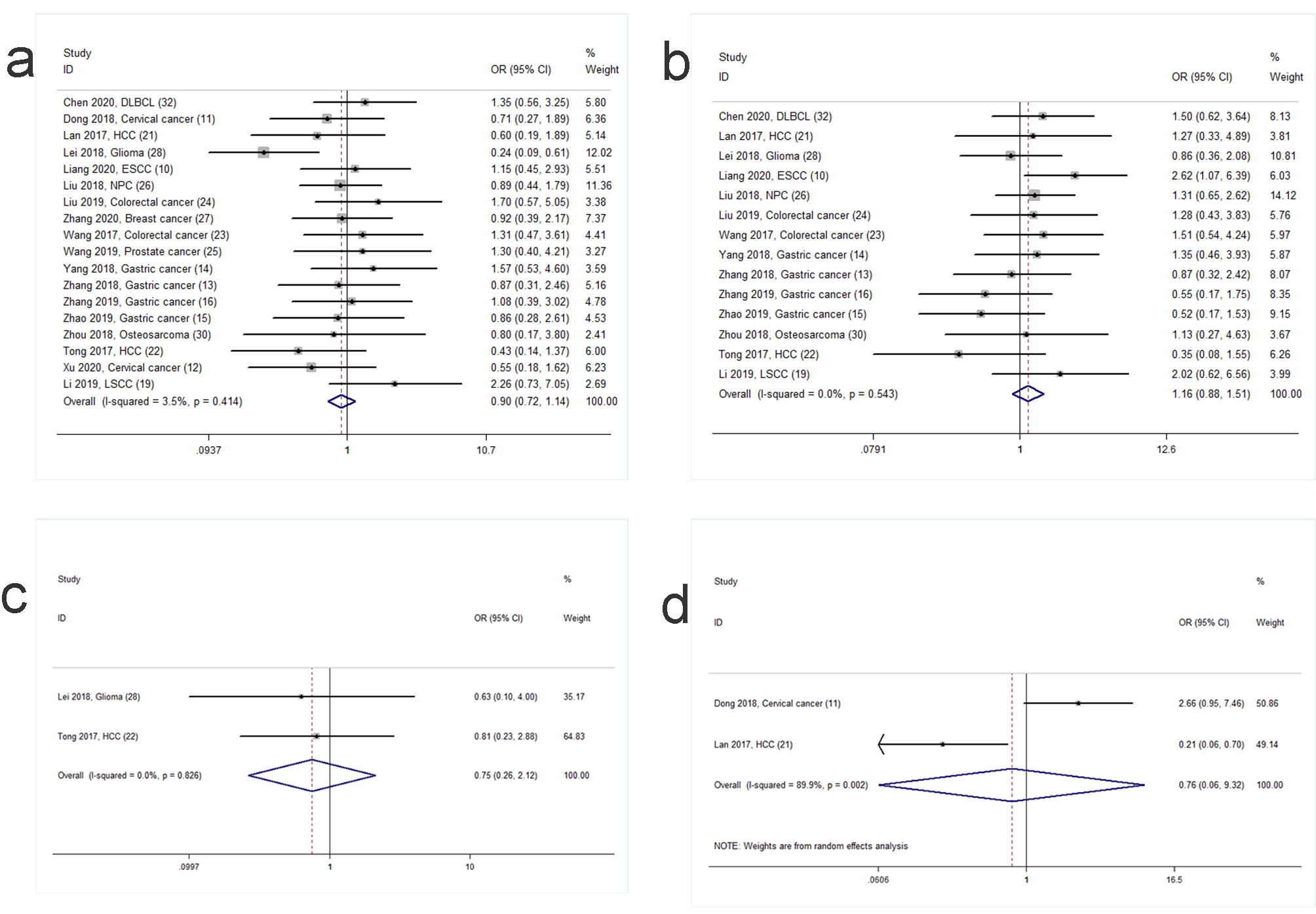

Supplement: Supplementary 1 — Supplementary Figure S1. Forest plots of studies evaluating odds ratios (ORs) of SNHG12 expression and the clinicopathology features, including (a) age, (b) gender, (c) tumor number, and (d) vascular invasion. Supplementary Figure S2: funnel plots (Begg's method) of potential publication bias for (a) age, (b) gender, (c) clinical stage, (d) lymph node metastasis, (e) differentiation grade, and (f) tumor size. [file 8847401.f1.zip › Supplementary Figure S1.jpg]

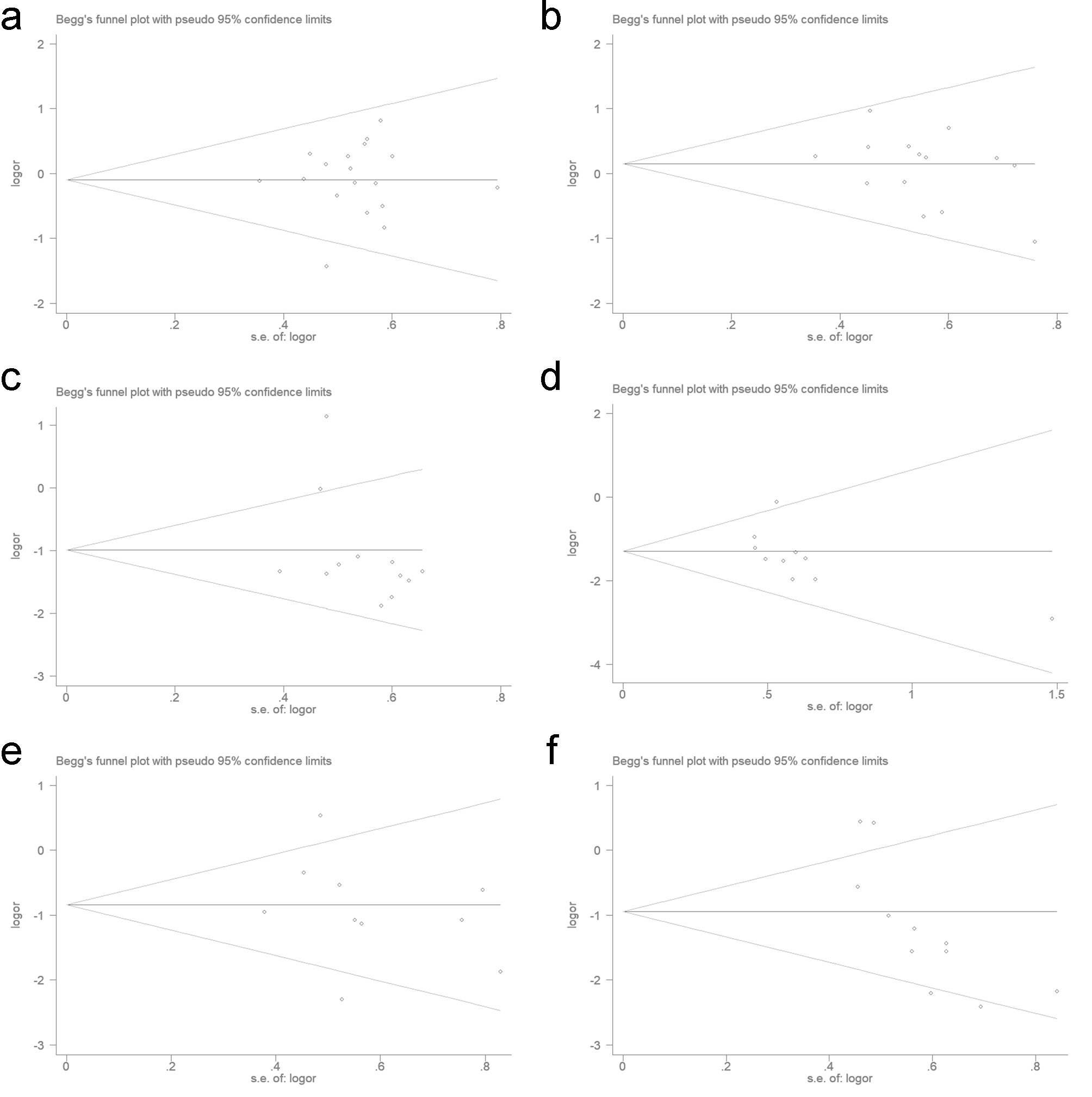

Supplement: Supplementary 1 — Supplementary Figure S1. Forest plots of studies evaluating odds ratios (ORs) of SNHG12 expression and the clinicopathology features, including (a) age, (b) gender, (c) tumor number, and (d) vascular invasion. Supplementary Figure S2: funnel plots (Begg's method) of potential publication bias for (a) age, (b) gender, (c) clinical stage, (d) lymph node metastasis, (e) differentiation grade, and (f) tumor size. [file 8847401.f1.zip › Supplementary Figure S2.jpg]
