# Supplementary material for: Prognostic Value of Long Noncoding RNA SNHG12 in Various Carcinomas: A Meta-Analysis
Source: Biomed Res Int. 2020 Nov 26;2020:8847401. doi: 10.1155/2020/8847401 (PMC7716752; doi:10.1155/2020/8847401)
Supplement: Supplementary 2 — Supplementary Table S1. The detailed example of the full electronic search strategy for PubMed. Supplementary Table S2: PRISMA Checklist. [file 8847401.f2.zip › Supplementary Table S1.docx]

Supplementary Table S1. The detailed example of the full electronic search strategy for PubMed

| Search | Query | Items found |
| --- | --- | --- |
| 3 | (((((long non-coding RNA small nucleolar RNA host 12) OR (long non-coding RNA SNHG12)) OR (SNHG12 lncRNA)) OR (lncRNA SNHG12)) OR (SNHG12)) AND (((((((((((((((((Neoplasia) OR Neoplasias) OR Neoplasm) OR Tumors) OR Tumor) OR Cancer) OR Cancers) OR Malignancy) OR Malignancies) OR Malignant Neoplasms) OR Malignant Neoplasm) OR Neoplasm, Malignant) OR Neoplasms, Malignant) OR Benign Neoplasms) OR Neoplasms, Benign) OR Benign Neoplasm) OR Neoplasm, Benign) | 75 |
| 2 | ((((((((((((((((Neoplasia) OR Neoplasias) OR Neoplasm) OR Tumors) OR Tumor) OR Cancer) OR Cancers) OR Malignancy) OR Malignancies) OR Malignant Neoplasms) OR Malignant Neoplasm) OR Neoplasm, Malignant) OR Neoplasms, Malignant) OR Benign Neoplasms) OR Neoplasms, Benign) OR Benign Neoplasm) OR Neoplasm, Benign | 4744317 |
| 1 | ((((long non-coding RNA small nucleolar RNA host 12) OR (long non-coding RNA SNHG12)) OR (SNHG12 lncRNA)) OR (lncRNA SNHG12)) OR (SNHG12) | 90 |
